# Growth promotion of *Spirulina* by steelmaking slag: application of solubility diagram to understand its mechanism

**DOI:** 10.1186/s13568-016-0270-4

**Published:** 2016-10-12

**Authors:** Reijiro Nogami, Haruo Nishida, Dang Diem Hong, Minato Wakisaka

**Affiliations:** 1 Graduate School of Life Science and Systems Engineering, Kyushu Institute of Technology, 2-4 Hibikino, Fukuoka, 808-0196 Japan; 2 Department of Algal Biotechnology, Institute of Biotechnology, Vietnam Academy of Science and Technology, 18 Hoang Quoc Viet Rd, Cau Giay Dist, Hanoi, Vietnam

**Keywords:** Steelmaking slag, Growth promotion, *Spirulina*, Solubility diagram, Oxidative stress

## Abstract

A solubility diagram was employed to understand growth promotion of *Arthrospira* (*Spirulina*) *platensis* by steelmaking slag (SMS). The growth promotion effect of 112 % of freshwater microalga *A. platensis* was obtained using 5 g/L SMS. However, metabolites, such as pigments, and protein content of *A. platensis* were not significantly affected. Several metals dissolved in Spirulina–Ogawa–Terui medium were detected by inductively coupled plasma atomic emission spectrometry just after the addition of SMS. The solubility diagram provides information on the chemical speciation of metal elements based on pH and concentration. It is a useful tool to understand the effect of metals on microalgal growth. The metal elements used to control microalgal growth are essential minerals but also act as a source of oxidative stress. Regarding the affecting mechanism of SMS, iron may be the primary element regulating microalgal growth via pathway involving reactive oxygen species, as revealed by superoxide dismutase assay.

## Introduction

Microalgae are potential sources of cosmetic, food and pharmaceutical products, and biofuels (Xi et al. 2016; Tasić et al. 2016; Santos et al. 2016). However, the productivity of microalgal cultures must be improved for scale-up application.

To improve the productivity of microalgal culture, various efforts, such as screening of better strain selection (Chen et al. 2009; Pereira et al. 2011; Chen et al. 2012; Bajhaiya et al. 2016) and optimization of culture conditions, have been made (Kim et al. 2012; Kanaga et al. 2016). The enhancement of microalgal growth and accumulation of high value products can be achieved by simply adding various chemical substances (Fábregas et al. 1987; Sasaki et al. 1995; Valenzuela-Enrique et al. 2002; Moed et al. 2015; Nayak et al. 2016). Steelmaking slag (SMS), a by-product of iron-making process, is an effective fertilizer for seaweed bed restoration (Takahashi and Yabuta 2002; Yabuta et al. 2006; Miyata et al. 2009; Hayashi et al. 2011; Yamamoto et al. 2012). SMS contains minerals, such like Fe, P, Mg, Ca, Mn, that are essential for algal growth (Yokoyama et al. 2010; Zhang et al. 2012; Mombelli et al. 2014). The growth promotion effect of SMS has been reported in not only macroalgae but also in certain seawater microalgae (Nakamura et al. 1998; Haraguchi et al. 2003; Sugie and Taniguchi 2011). However, investigations on freshwater microalgae required for the commercial production of valuable products are fewer than those on marine microalgae. In our previous study, we demonstrated growth promotion effect of SMS to *Spirulina*, a freshwater microalgae (Nogami 2016). However, the growth promotion mechanism of SMS is still unclear. Although there are current prevailing opinions, such as the fertilization effect of eluted iron necessary for photosynthesis (Yamamoto et al. 2016) or the contribution of dissolved CO_2_ via increase in the pH of the medium (Takahashi et al. 2012), there are no unified views to explain growth promotion effect of SMS.

On the other hand, the dissolution behavior of various elements from SMS in seawater and freshwater were demonstrated using solubility diagram (Futatsuka et al. 2004; Miki et al. 2004; Yokoyama et al. 2012). However, there are no reports on the application of a solubility diagram of SMS for understanding microalgal growth profile. The solubility diagram provides information on the stable chemical speciation under different concentration and pH and is useful for understanding the elution or precipitation behavior of solutions. SMS is a mixture of various metal elements. The elution behavior of each element from SMS to culture medium and microalgal growth profile should be correlated to understand the mechanism underlying the growth promotion effect. Elements eluted from SMS to culture medium can be detected using atomic absorption spectrometry to understand the effect on microalgal culture.

This study aimed to understand the correlation between microalgal growth and speciation of metals eluted from SMS using a solubility diagram obtained for *Spirulina* culture with SMS.

## Materials and methods

### Materials

The cyanobacterium *Arthrospira platensis* (Nordstedt) Gomont NIES-39 strain was purchased from the National Institute for Environmental Studies, Tsukuba, Japan. *A. platensis* was cultured in 300 mL flasks containing 200 mL of Spirulina–Ogawa–Terui (SOT) medium with the following composition (mg L^−1^): NaHCO_3_, 16,800; K_2_HPO_4_, 500; NaNO_3_, 500; K_2_SO_4_, 1000; NaCl, 1000; MgSO_4_·7H_2_O, 200; CaCl_2_·2H_2_O, 40; FeSO_4_·7H_2_O, 10; Na_2_EDTA·2H_2_O, 80; H_3_BO_3_, 2.86; MnSO_4_·5H_2_O, 2.5; ZnSO_4_·7H_2_O, 0.22; CuSO_4_·5H_2_O, 0.08; and Na_2_MoO_4_·2H_2_O, 0.02. SMS, 2 mm in diameter, was added to SOT medium at 0, 0.05, 0.5, and 5 g L^−1^ in microalgal cultures and blanks. *A. platensis* was cultivated under the following conditions: a light intensity of 12,000 Lux from a white fluorescent lamp, with 12 h/12 h light/dark cycles and a temperature of 25 °C. All flasks were cultured under static conditions and were shaken by hand twice a day.

### Cell growth and metabolite analysis

Cell growth was determined by measuring the dry weight of the biomass. Cells were filtered using filter paper (GC-50, ADVANTEC), oven dried at 105 °C for 2 h, and were placed in a desiccator for 1 h before measuring the weight. Biomass weight was calculated by subtracting the dry weight of the blank. Chlorophyll a and phycocyanin were repeatedly extracted using 80 % acetone and 0.01 M potassium phosphate buffer of pH 7.8, respectively. Pigment contents was calculated by measuring their absorbance at 750 nm using spectrophotometer (UV–vis 1200, Shimadzu). Protein was extracted by salting out, and its concentration was determined by Lowry et al. (1951).

### Solubility diagram

The culture medium was sampled every 7 days to monitor pH and metal elution from SMS. After filtering the culture medium, the pH of the filtrate was measured using a pH meter (LAQUA twin, Horiba), and metal elution was detected using Simultaneous ICP Atomic Emission Spectrometer (ICPE-9800, Shimadzu). The solubility diagram of eluted elements was obtained based on the calculation of solubility product and chemical potential (Futatsuka et al. 2004).

### SOD activity assay

Superoxide dismutase (SOD) activity was measured to determine the microalgal response to metals eluted from SMS. Microalgal cells were harvested by centrifugation and homogenized with potassium phosphate buffer. The homogenates were then centrifuged at 12,000 rpm for 10 min at 4 °C. Xanthine oxidase was used to generate O_2_^−^, and SOD assay was followed.

### Statistical analysis

All experiments were performed in triplicates, and the data has been presented as mean ± standard deviation. Data were statistically analyzed using Kruskal–Wallis test, with the level of significance at p < 0.05.

## Results

### Growth promotion of *A. platensis* by SMS

**Fig. 1:**
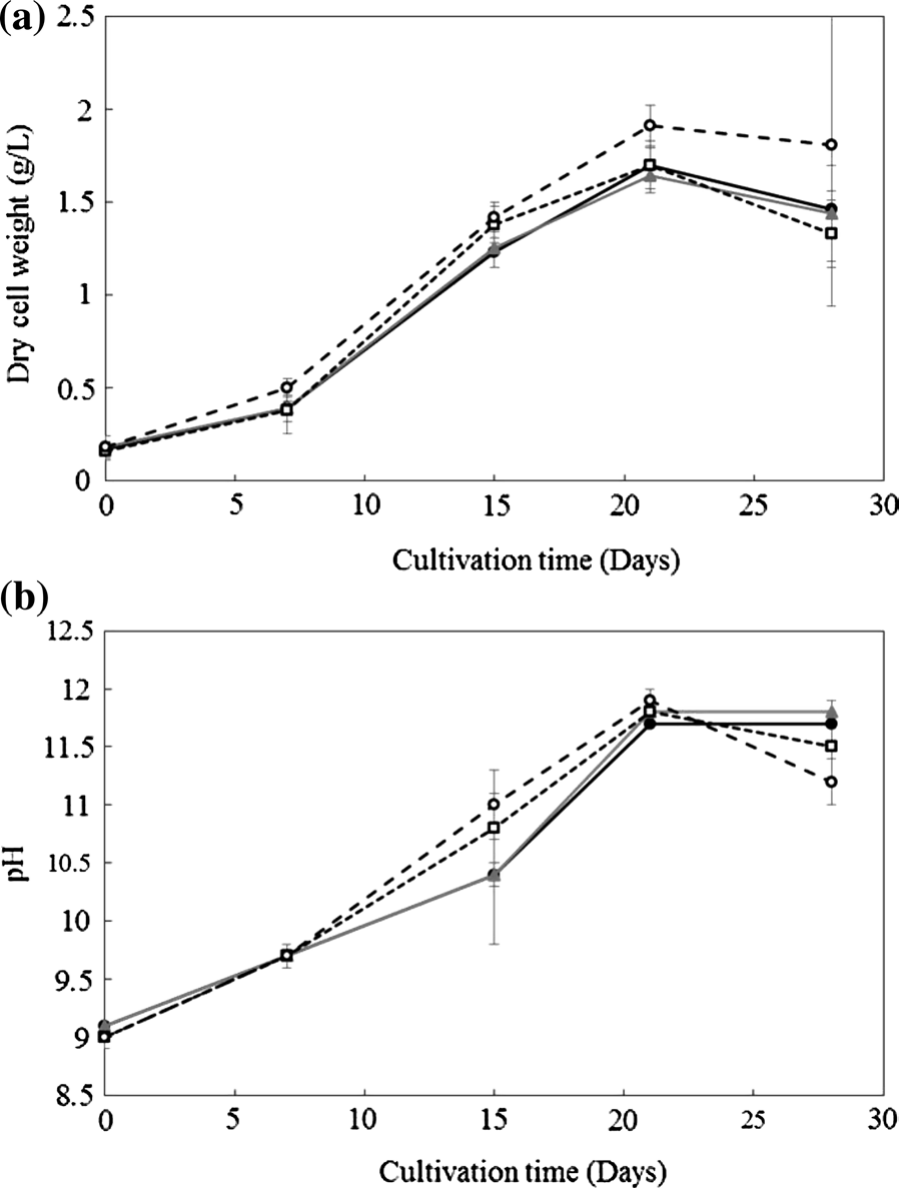
Effects of steelmaking slag on *Arthrospira platensis* culture. **a** Growth profile, **b** pH change during culture. (●) Control, (▲) 0.05 g/L SMS, (□) 0.5 g/L SMS, (○) 5 g/L SMS

The growth promotion effect of SMS on the freshwater microalga *A. platensis* was comparable to that of other marine microalgae, which was previously reported. Figure 1a shows the growth profile of *A. platensis*. Its growth significantly increased by the addition of 5 g/L SMS during the latter half of culture. The maximum growth promotion of 1.12-fold higher than that of control was obtained at 21 days. Figure 1b shows the pH change during culture period. pH increased corresponding to the increase in culture time until 20 days, when maximum growth was observed that stabilized at pH 11.5 in control and with 0.05 g/L SMS and slightly decreased below pH 11.5 with 0.5 and 5 g/L SMS. The trend in increase in growth differed based on the amount of SMS, i.e., it proportionally increased with 0.5 and 5 g/L SMS, with rapid increase observed after 15 days in control and with 0.05 g/L SMS.

### Metabolite analysis of *A. platensis*

**Fig. 2:**
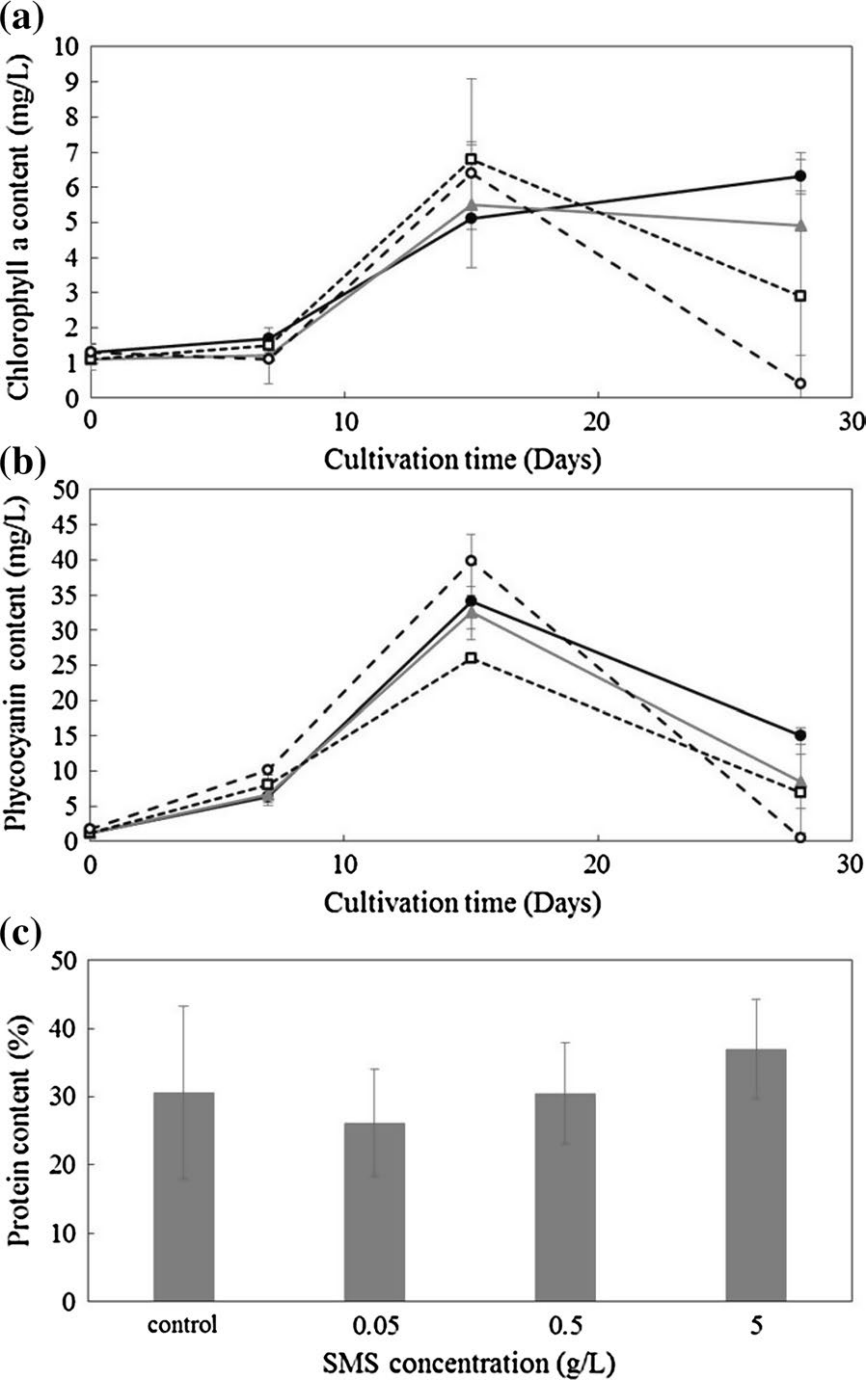
Effects of steelmaking slag on the metabolites of *Arthrospira platensis*. **a** Chlorophyll a, **b** phycocyanin, **c** protein content of (●) Control, (▲) 0.05 g/L SMS, (□) 0.5 g/L SMS, (○) 5 g/L SMS

Metabolites, such as pigment, and protein content of *A. platensis* were not significantly affected by SMS. Figure 2 shows pigment contents [(a) chlorophyll a, (b) phycocyanin] of *A. platensis*. Pigment contents did not significantly differ until 14 days, but at the end of culture, the pigment contents were decreased in SMS concentration-dependent manner compared with those of the control. There was no significant difference in total protein content observed as shown in Fig. 2c.

### Metal elution from SMS to culture medium

**Fig. 3:**
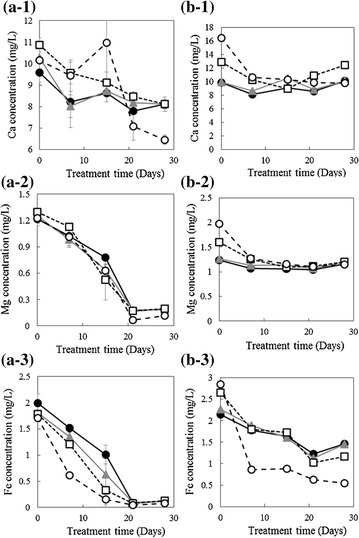
Elemental dissolution from steelmaking slag (SMS) in Spirulina–Ogawa–Terui medium. **a** Behavior with *Arthrospira platensis* (a-1: Ca, a-2: Mg, a-3: Fe), **b** Behavior without *A. platensis* (b-1: Ca, b-2: Mg, b-3: Fe), of (●) Control, (▲) 0.05 g/L SMS, (□) 0.5 g/L SMS, (○) 5 g/L SMS

The dissolution of metals in SOT medium was detected using inductively coupled plasma atomic emission spectrometry just after the addition of SMS. Figure 3 shows the concentrations of the dissolved metals in SOT medium compared between samples with and without microalgal cells. The dissolution of Ca, Mg, and Fe from SMS was evident at 0 day without microalgal cells, and the concentrations of all dissolved metals decreased with the increase in microalgal cells. In the case with microalgal cells, the concentrations of dissolved Ca, Mg, and Fe decreased over time, but trends in this decrease differed for each metal. The concentration of dissolved Mg sharply decreased to almost zero after 15 days. Change in Fe concentration was observed in SMS concentration-dependent manner. The concentration of dissolved Fe exhibited a more severe decrease after 15 days in the control without SMS than in that with SMS. Samples without microalgal cells exhibited initial decreases in the Ca and Mg concentrations, which subsequently stabilized; However, Fe concentration exhibited a decreasing trend with 5 g/L SMS.

### Solubility diagram applied for metal elution from SMS

**Fig. 4:**
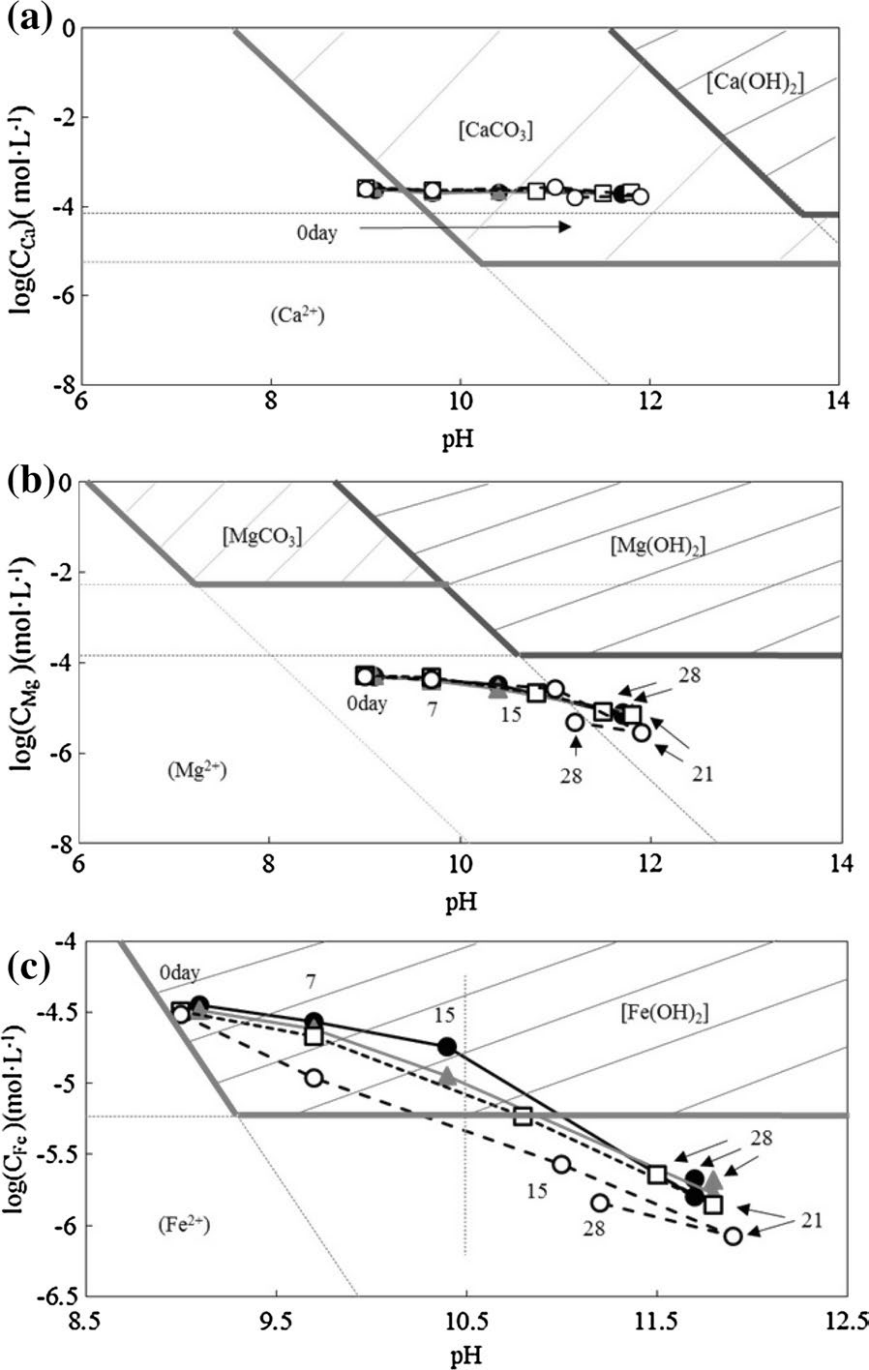
Solubility diagram of each element applied for *Arthrospira platensis* culture. Solubility diagram of **a** Ca, **b** Mg, and **c** Fe for (●) Control, (▲) 0.05 g/L SMS, (□) 0.5 g/L SMS, (○) 5 g/L SMS

The solubility diagram is a useful tool for understanding the chemical speciation of metals and thus their bioavailability during microalgal culture. Figure 4 shows the solubility diagram of Ca, Mg, and Fe for *A. platensis* culture. Correlation between the concentration of dissolved metal and pH during culture period was plotted in each solubility diagram. Ca precipitated as CaCO_3_ soon after elution from SMS in the SOT medium during the culture period, whereas Mg and Fe changed to their respective hydroxides. Fe was considered to be the primary element controlling growth or *A. platensis* since highest decrease of concentration among three elements was observed.

### Oxidative stress by Fe and growth control of *A. platensis*

**Fig. 5:**
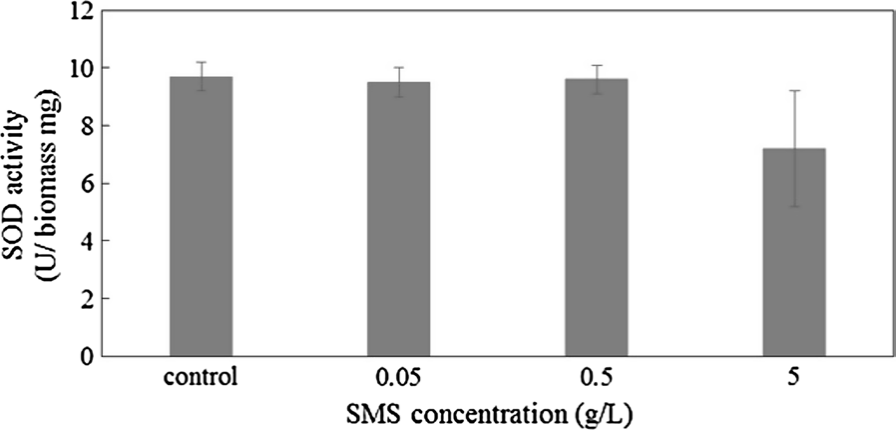
Superoxide dismutase activity of *Arthrospira platensis* exposed to steelmaking slag

To understand the growth control mechanism of Fe from SMS, the biological response to oxidative stress by metal elution from SMS was investigated. Figure 5 shows the SOD activity of *A. platensis* at 21 days after exposure to SMS. The SOD activity of 5 g/L SMS significantly decreased corresponding to the considerable decrease in Fe concentration compared with that of the other SMS concentrations.

## Discussion

The growth profile results (Fig. 1a) are consistent with the reported values (Carvalho et al. 2004; Chen et al. 2006; Göksan et al. 2007; Markou et al. 2012). This could be due to initial pH of 9.0 achieved by adding SMS was optimal for *A. platensis* (Fig. 1b).

*A. platensis* pigment contents in SMS decreased compared with those in the control at the end of culture (Fig. 2a, b). This may be explained by the pH dependency of pigments, with a reported maximum content at pH 8.5 for chlorophyll a and 9.0 for phycocyanin (Ismaiel et al. 2016). Another reason could be due to iron deficiency. The decrease in pigment contents was also reported with another type of cyanobacterium, *Aphanocapsa* (Sandmann 1985).

Changes in the concentration of each concentration in Fig. 3 may be explained by iron hydroxide precipitation followed by calcium hydroxide formation. Thus, Fe eluted from SMS is considered as the primary element controlling microalgal growth. The solubility diagram, which provides information on the chemical speciation of each elements based on to pH and concentration provides insight on this.

**Fig. 6:**
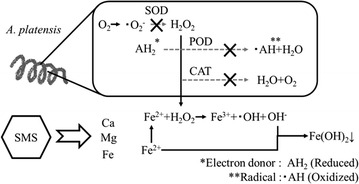
Schematic illustrations of the biological mechanism of *Arthrospira platensis* respond to steelmaking slag

The results of SOD activity (Fig. 5) were consistent with the results in another report which stated that SOD activity is dependent on iron concentration (Ismaiel et al. 2014). Enzymatic activity involving antioxidation, such as that of catalase (CAT), peroxidase (POD), and SOD, of *A. platensis* is known to be dependent on pH level. Optimal pH of 9 was reported for CAT, 10 for POD, and 10.5 for SOD (Ismaiel et al. 2016). The antioxidative capacity of *A. platensis* supposedly diminished due to increase in pH during the culture period. Reactive oxygen species resulting from this lack of antioxidative capacity reacted with Fe ion eluted from SMS, which is thought to result in the changes in concentration and speciation. Thus, we established a hypothetic scheme to comprehensively understand the correlation between *A. platensis* growth, Fe speciation and pH, as shown in Fig. 6.

Firstly, enzymatic activity relating to antioxidation, such as that of POD, CAT, and SOD, decreased in this order corresponding with the increase in pH during culture. Subsequently, hydrogen peroxide, not thoroughly treated by antioxidative enzymes, leaked out from the cell into the culture medium. Fenton reaction occurred between hydrogen peroxide and ferrous ion eluted from SMS to produce hydroxide ion and hydroxyl radical, which damages the cells. Finally, hydroxide ion reacted with ferrous iron and precipitated as iron hydroxide. This hypothesis explains the experimental results found by adding 5 g/L SMS, which exhibited maximum growth, iron depletion, and loss of SOD activity due to the increase in pH at 21 days. The application of solubility diagrams contributes to the understanding of microalgal growth regulation mechanism in aquatic condition.

## Abbreviations

SMS: steelmaking slag
SOT: Spirulina–Ogawa–Terui
SOD: superoxide dismutase
CAT: catalase
POD: peroxidase
